# Coach-Assisted eHealth With Group or Individual Support for Employees With Obesity: Randomized Controlled Trial on Weight, Body Composition, and Health Metrics

**DOI:** 10.2196/60436

**Published:** 2025-03-12

**Authors:** Siniriikka A Männistö, Kirsi H Pietiläinen, Joona Muotka, Laura-Unnukka Suojanen, Raimo Lappalainen, Riitta Korpela

**Affiliations:** 1 Occupational Health Helsinki Helsinki Finland; 2 HealthyWeightHub, Endocrinology, Abdominal Center, Helsinki University Hospital and University of Helsinki Helsinki Finland; 3 Obesity Research Unit, Research Program for Clinical and Molecular Metabolism, Faculty of Medicine, University of Helsinki Helsinki Finland; 4 Department of Psychology University of Jyväskylä Jyväskylä Finland; 5 Department of Pharmacology Faculty of Medicine University of Helsinki Helsinki Finland

**Keywords:** eHealth, weight loss, acceptance and commitment therapy, weight-neutral, Healthy Weight Coaching, occupational health, digital health, body composition, obesity, psychobehavioral, intervention, health care, metabolic health, physiological change

## Abstract

**Background:**

Acceptance and commitment therapy provides a psychobehavioral framework feasible for digital and hybrid weight loss interventions. In face-to-face studies, group-based interventions yield more favorable outcomes than individual interventions, but the effect of the intervention form has not been studied in combination with eHealth.

**Objective:**

This study investigated whether a minimal, 3-session group or individual enhancement could provide additional benefits compared to an eHealth-only intervention when assessing weight, body composition, and laboratory metrics in a sample of occupational health patients with obesity.

**Methods:**

This study was a randomized controlled trial with a 12-month intervention, followed by a 12-month follow-up period without additional support (March 2021 to March 2023). Recruited from occupational health care for Finnish municipal employees, 111 working-age adults with a BMI of 30-40 kg/m^2^ were randomized to 1 of the 3 treatment arms: eHealth, eHealth+group, or eHealth+individual. All treatment arms received a web-administrated, coach-assisted eHealth program based on acceptance and commitment therapy, and additionally, the eHealth+group and eHealth+individual arms received 3 remotely facilitated group or individual meetings with their designated coach. The participants were assessed for weight, body composition, blood pressure, and laboratory measurements at 0-, 6-, 12-, and 24-month time points. Applying estimated means to decrease bias caused by dropouts, generalized estimating equations were used to study the differences between the 3 groups over time.

**Results:**

There were no between-group differences in primary measurements of weight change or categorical weight change. Secondary outcomes also did not show changes attributable to the intervention arm. Across the entire sample, the total weight loss was 1.5% during the intervention, with 18% (20/111) of the participants attaining a ≥5% weight loss. Sustained at follow-up, waist circumference decreased, and high-density lipoprotein cholesterol increased slightly. The participants completed, on average, 58.6% of the eHealth program.

**Conclusions:**

There were no differences in weight or other somatic health variables between the eHealth arm and intervention combining eHealth with minimal group or individual enhancement. Despite a modest overall weight loss, the intervention shows promise in improving body composition and metabolic health. Moving forward, further research is needed to determine if there is a threshold where face-to-face meetings provide additional benefits in hybrid interventions. Moreover, there is a need to explore for whom and under what conditions eHealth and hybrid models may be most effective.

**Trial Registration:**

ClinicalTrials.gov NCT04785586; https://clinicaltrials.gov/study/NCT04785586

## Introduction

The prevalence of obesity has more than doubled worldwide since the 1970s [1]. Obesity increases the risk for several metabolic, mechanical, and psychiatric conditions, attributing to 8% of deaths worldwide [2,3]. Weight loss is seen to effectively alleviate obesity-related comorbidities, and even a modest 5%-10% weight loss is seen to, for example, lower hypertension and total cholesterol, and prevent the onset of diabetes [4-6].

However, attaining even modest weight loss results is not an easy task because weight is seen to increase by 1-2 kg yearly after weight loss intervention [7]. Research has demonstrated the significant role of psychobehavioral factors in weight management success, leading to the use of psychological components in weight loss interventions [8,9]. Acceptance and commitment therapy (ACT) is a third-wave cognitive behavioral therapy aiming to increase psychological flexibility—defined by Hayes et al [10] as “the ability to contact the present moment more fully as a conscious human being, and to change or persist in behavior when doing so serves valued ends.” ACT promotes engagement in health-promoting behavior patterns while enhancing commitment to values-based behaviors [11]. Although ACT primarily targets psychological well-being rather than direct weight loss, it has proven effective in supporting long-term weight management and lifestyle changes [12,13].

In the context of weight loss trials, ACT is usually delivered in a group or workshop format [14-20]. However, in recent years, there has been growing attention to digital interventions for weight loss, offering an accessible, widespread, and cost-effective alternative to traditional face-to-face treatments [21,22]. Indeed, recent systematic reviews have provided compelling evidence supporting the effectiveness of digital interventions (eHealth) in obesity treatment: without distinguishing between different theoretical frameworks, eHealth interventions have demonstrated outcomes on par with traditional face-to-face methods and have consistently outperformed control conditions [23].

Adding personalized human interaction and guidance to eHealth programs has proven to be particularly effective in achieving significant weight loss [24-27]. Furthermore, combining face-to-face meetings with digital interventions, referred to as hybrid interventions, has been shown to further enhance the effectiveness of weight loss [28-30]. ACT-based hybrid interventions are still rare, but studies combining digital ACT interventions with group meetings [31] or telephone support [32,33] have shown promising results. The impact of intervention format (group vs individual) on weight loss has not been studied in combination with eHealth, particularly in the context of ACT. However, in face-to-face trials, group-based interventions have consistently produced more favorable outcomes compared to individual approaches [34,35].

This study aimed to investigate the differences in the ACT-based 12-month eHealth intervention between eHealth, eHealth+group, and eHealth+individual delivered for occupational health patients with a further 12-month follow-up period. Weight and categorical weight change were considered the primary outcomes, and waist circumference, body composition, and laboratory measurements were the secondary outcomes of this study. We hypothesized that enhancing interventions with individual or group meetings alongside the eHealth program would lead to improved weight and health outcomes, particularly favoring the group treatment arm. Additionally, we anticipated significant changes in weight and health parameters across all treatment groups.

## Methods

### Study Design

This study was a randomized controlled trial with a 12-month intervention and 12-month follow-up period. The participants were allocated to 1 of the 3 treatment arms: eHealth, eHealth+group, or eHealth+individual treatment in a 1:1:1 ratio. All treatment arms received a web-administrated eHealth program, and additionally, the eHealth+group or eHealth+individual arms received 3 remotely facilitated group or individual meetings.

The eHealth program used in this study, Healthy Weight Coaching (HWC), is a 1-year treatment program based on the ACT framework, aiming to increase psychological flexibility and teach skills related to weight management [36]. The program consists of web-administrated weekly exercises integrating ACT with behavioral weight management themes (eating behavior, physical activity, sleep, and stress; Table S1 in Multimedia Appendix 1). The HWC program is readily available in Finnish public health care, and this study was designed to investigate whether minimal enhanced support in a group or individual format could improve attrition and response to the treatment.

The version of the program used in this study adopted a softer, more weight-neutral coaching style than the HWC protocol, with prioritization on value-based actions, mindfulness, and health-promoting changes instead of weight loss itself. The enhanced group and individual meetings in eHealth+group and eHealth+individual arms adhered closely to the ACT framework, incorporated key lifestyle change components of the program, and aimed to deepen the participants’ understanding of the program’s themes.

The participants were each assigned a personal coach who provided periodic, tailored, one-on-one written feedback and facilitated any additional meetings. All the 3 coaches in this study had master’s degrees in nutrition and further education in psychology. They met biweekly to ensure adherence to this study’s protocol. The coaches worked across the 3 treatment arms, being randomly assigned to one-third of each.

### Treatment Arms

- eHealth: Participants received the HWC eHealth program, which included a 20-minute kickoff call 3 weeks into the program to ensure they were progressing as expected. They also received written feedback every 2 weeks for the first 3 months and then once every 3 weeks for the remainder of the year, totaling 19 contacts.
- eHealth+group: Participants received the HWC eHealth program (as described in item 1, eHealth) and, in addition, participated in 3 remotely facilitated group meetings using video (each was 2 hours long; due to COVID-19, a transition to remote meetings was necessitated). These group meetings included brief introductions to covered topics (eg, goal setting, stress management, and self-compassion) followed by small group discussions (Table S2 in Multimedia Appendix 1). The group meetings occurred at 1, 6, and 10 months into the 12-month treatment period.
- eHealth+individual: Participants received the HWC eHealth program (as described in item 1, eHealth) and, in addition, participated in 3 remotely facilitated individual meetings using video (each was 45 minutes long). These individual meetings were customized to each participant’s current needs, acknowledging successes and seeking solutions to challenging situations. The individual meetings occurred at 1, 6, and 10 months into the 12-month treatment period.

### Recruitment

Participants were Helsinki city employees with class 1 and class 2 obesity [37]. Recruitment was conducted by the staff of the occupational health care and through recruitment advertisements on the city employees’ communication platforms. Enrollment was carried out by research assistants from February to March 2021. Inclusion criteria required participants to have a BMI between 30 to 40 kg/m^2^, the ability to participate in group or individual meetings, access to a computer with internet services, and fluency in the Finnish language. Exclusion criteria included participation in other weight loss programs, pregnancy or lactation within the past 6 months, weight changes exceeding 5 kg within the past 3 months, major medical conditions affecting safety or readiness for weight loss, and the use of weight loss medication.

### Power Analysis and Sample Size

The sample size of 23 participants per treatment arm was determined by a statistician (JM) with priori power analysis using a statistical power of 0.80 with 2-sided test and 0.05 α, modeling the mean weight change and SD in previous weight loss studies (mean 3.8, SD 4.6 kg) [38]. With an expected attrition rate of 40%, the target number of participants was set at 38 per treatment arm.

### Screening

Following recruitment, participants underwent a health screening on a phone by a medical doctor covering their health status and medications. Those who passed the screening were then referred for laboratory assessments and body composition measurements.

### Randomization

In March 2021, participants who provided written consent and completed baseline measurements were randomized into 1 of the 3 treatment arms (see figure in the Results section). Randomization was carried out by a statistician (JM) using stratified random allocation with SPSS (IBM Corp). Participants were stratified by sex (male or female), age groups (20-35, 35-50, and 50-65 years), and BMI categories (≥30 to 35 or ≥35 to 40 kg/m^2^).

### Outcome Measures

The intervention period lasted for 12 months, from March 2021 to March 2022, after which a 12-month follow-up period was conducted until March 2023 without further support. Participant assessments were conducted at baseline, midintervention (6 months), postintervention (12 months), and follow-up (24 months) at Occupational Health Helsinki. The following measurements were taken:

- Height: Measured to the nearest 0.1 cm using a Gima tape height measure with a scale from 0-200 cm (Gima SpA).
- Weight: Measured after an overnight fast to the nearest 0.1 kg in light clothing, barefoot, on InBody720 or InBody770 (InBody Co Ltd), with repeated measures taken using the same equipment.
- Body composition: Fat mass, muscle mass, and visceral fat were measured with the abovementioned InBody devices.
- Waist circumference: Measured to 0.1 cm using a Hoechstmass nonextensible tape measure (Hoechstmass Balzer GmbH) at the narrowest point between the lower costal border and the umbilicus.
- Blood pressure and heart rate: Systolic and diastolic blood pressure and heart rate were measured 3 times after 10 minutes of rest using an Omron automated blood pressure monitor (Omron Corporation). The mean sum of the last 2 measurements was used in the analysis, and the measurements were repeated if necessary until the difference between the last 2 measurements was <10 mm Hg.
- Blood samples: Blood samples were collected at the laboratory of Occupational Health Helsinki after an overnight fast and analyzed for lipids (total cholesterol, low-density lipoprotein cholesterol, high-density lipoprotein [HDL] cholesterol, and triglycerides), glucose, hemoglobin A1c (HbA1c), alanine transaminase, and high-sensitivity C-reactive protein (hs-CRP).

Of the baseline characteristics (Table 1), metabolic syndrome was calculated from the laboratory and anthropometric measurements following the International Diabetes Foundation’s 2006 definition [39]. Smoking was self-reported in a baseline survey. The professional role was determined using Statistics Finland’s Classification of Occupations 2010 index [40]. Program usage was obtained from the log file. Medication changes were tracked every 3 months as part of the eHealth program.

**Table 1:** Baseline characteristics of participants by treatment arm and across the entire sample.

Characteristic				Treatment arm									
				eHealth (n=38)		eHealth+group (n=35)		eHealth+individual (n=38)		Total (N=111)	*P* value^a^		
**Age (years)**													
	Mean (SD)		52.3 (9.7)		50.6 (7.9)		49.6 (8.8)		50 (8.9)		.41		
	**Age group, n (%)**										.91		
		<35	2 (5)		2 (6)		3 (8)		7 (6)				
		35-50	11 (29)		12 (34)		14 (37)		37 (33)				
		>50	25 (66)		21 (60)		21 (55)		67 (60)				
**Sex, n (%)**													.53
	Male		8 (21)		4 (11)		7 (18)		19 (17)				
	Female		30 (79)		31 (89)		31 (82)		92 (83)				
**Smoking status, n (%)**													.54
	Never smoker		19 (50)		14 (40)		17 (45)		50 (45)				
	Former smoker		18 (47)		15 (43)		16 (42)		49 (44)				
	Current smoker		1 (3)		4 (11)		5 (13)		10 (9)				
**Comorbidities, n (%)**													
	Metabolic syndrome		28 (74)		20 (57)		25 (66)		73 (66)		.33		
	Type 2 diabetes		1 (3)		4 (11)		2 (5)		7 (16)		.29		
	Sleep apnea		8 (21)		5 (14)		2 (5)		15 (14)		.13		
	Mental health problem		8 (21)		5 (14)		10 (26)		23 (21)		.45		
	Osteoarthritis		7 (18)		9 (26)		5 (13)		21 (19)		.39		
**Professional role, n (%)**													.79
	Managers		0 (0)		2 (6)		2 (5)		4 (4)				
	Specialized experts		15 (40)		9 (26)		10 (26)		34 (31)				
	Experts		14 (37)		17 (49)		15 (39)		46 (41)				
	Office and customer service workers		4 (11)		4 (11)		5 (13)		13 (12)				
	Service and sales workers		5 (13)		2 (6)		5 (13)		12 (11)				
	Construction, repair, and manufacturing workers		0 (0)		1 (3)		1 (3)		2 (2)				
**Anthropometric measurements, mean (SD)**													
	Height (cm)		168.9 (10.4)		165.5 (7)		167.1 (8.4)		167.2 (8.8)		.26		
	Weight (kg)		97.6 (12.8)		94.1 (11)		95.8 (12.9)		95.9 (12.3)		.47		
	BMI (kg/m^2^)		34.2 (2.7)		34.3 (2.9)		34.2 (3.1)		34.2 (2.9)		.98		
	Waist circumference (cm)		112.1 (8)		111.5 (8.7)		109.9 (9.7)		111.2 (8.8)		.53		
**Body composition, mean (SD)**													
	Visceral fat (cm^2^)		202.8 (37.8)		200.5 (31.7)		202.1 (36.1)		201.8 (35.1)		.96		
	Fat percentage (%)		42.9 (6.5)		43.3 (5.2)		43.4 (6)		43.2 (5.9)		.92		
	Muscle percentage (%)		31.8 (4.1)		31.5 (3.2)		31.5 (3.7)		31.6 (3.7)		.92		
**Laboratory measurements, mean (SD)**													
	Hemoglobin A_1c_ (mmol/mol)		38.4 (4.8)		38.6 (7.3)		39.8 (10.1)		38.9 (7.7)		.71		
	Glucose (mmol/L)		6.1 (0.8)		5.9 (0.9)		6.3 (2.1)		6.1 (1.4)		.55		
	Cholesterol (mmol/L)		5.3 (1.1)		5.4 (1.1)		5.3 (1)		5.4 (1)		.84		
	High-density lipoprotein (mmol/L)		1.3 (0.4)		1.3 (0.3)		1.3 (0.3)		1.3 (0.3)		.81		
	Low-density lipoprotein (mmol/L)		3.1 (0.8)		3.4 (1)		3.3 (0.8)		3.3 (0.9)		.25		
	Triglycerides (mmol/L)		2.1 (1.4)		1.6 (0.6)		1.4 (0.7)		1.7 (1)		.006		
	Alanine aminotransferase (U/L)		43.4 (36.1)		29.2 (14.4)		34.7 (17)		36 (25.2)		.05		
	High-sensitivity C-reactive protein (mg/L)		2.9 (2.1)		2.8 (2.5)		2.8 (2.5)		2.8 (2.4)		.96		
**Blood pressure**													
	Systolic (mm Hg)		129.7 (14.5)		127.3 (11.5)		126.7 (14.2)		127.9 (13.4)		.60		
	Diastolic (mm Hg)		88.4 (8.3)		86.8 (8)		86.6 (9.2)		87.3 (8.5)		.59		
	Pulse (beats per minute)		72.4 (10.4)		69.4 (9.2)		72.5 (8.9)		71.5 (9.5)		.30		

^a^*P* values indicate the difference between the 3 treatment arms with the categorical variables analyzed by chi-square test and the continuous variables by ANOVA.

### Statistical Analysis

Data analysis was conducted using IBM SPSS Statistics (version 28.0.0.0; IBM Corp). Baseline differences were analyzed by chi-square for the categorical variables and ANOVA for the continuous variables. Differences in mean values between the measurement points and the treatment arms were analyzed using the generalized estimating equations (GEEs) method [41]. The GEE method includes all the available data points and uses estimated means in the analysis, applying the intention-to-treat (ITT) analysis concept to decrease bias caused by dropouts. The article only reports results from the ITT analysis, while completer analyses are available in Multimedia Appendix 1.

Considering GEEs, we first used a model examining the interaction between the measurement points and treatment arms, with post hoc analysis comparing the effects of the eHealth+group and the eHealth+individual arms to the eHealth arm. For further analysis, we used a single-arm model, concentrating on the changes observed across the 4 measurement points in the entire study sample. After the primary analysis, we added baseline weight, sex, age, and applicable medication or medication change (eg, blood pressure medication in systolic and diastolic blood pressure analysis) to the single-arm model to explain the results. The changes in medication are described in Table S3 in Multimedia Appendix 1. Weight, BMI, waist circumference, HbA_1c_, cholesterol, HDL, low-density lipoprotein, systolic blood pressure, diastolic blood pressure, and pulse rate were normally distributed. Glucose, triglycerides, alanine transaminase, hs-CRP, and muscle percentage had a positively skewed distribution, and thus, gamma distribution with log link function was used in the GEEs analysis. Visceral fat and fat percentage had a negatively skewed distribution, and thus, inverted variables were used in the analysis with gamma distribution and log link function.

Crosstabs with Pearson chi-square was used when analyzing the group level differences of categorical weight variable (participants were divided into 6 categories based on weight loss percentage: <0, 0-2.9, 3-4.9, 5-5.9, 5-9.9, or ≥10), and change in metabolic syndrome prevalence between the measurement points. Categorical weight loss and metabolic syndrome prevalence are reported for the entire baseline sample, with the last observation carried forward. Changes in muscle and fat percentage, as well as metabolic syndrome incidence, are expressed as percentage points (%-point), describing the arithmetic difference between the 2 percentages.

Spearman correlation coefficient was used to analyze correlations between the weight change percentages (nonparametric). Dancey and Reidy’s definition was used when determining the strength of the correlation [42].

Cohen *d* effect size was calculated using the square root of the average variance of measures. In the analysis comparing the treatment arms, we used an effect size calculator developed by the statistician (JM) that adjusts for baseline differences. For the single-arm analysis, we used the University of Colorado Springs Effect Size Calculator [43].

Figures were drawn with GraphPad Prism (GraphPad Software).

### Ethical Considerations

This study’s protocol was approved by the Ethics Committee of the Helsinki and Uusimaa Hospital District (April 29, 2020, HUS/922/2020) and conducted per the ethical standards of the Declaration of Helsinki. Informed consent was obtained from all individuals included in this study. To ensure privacy and confidentiality, study data were pseudonymized and securely processed, preventing any association with individual participants. Participants received the intervention and individual health data but received no additional compensation. Neither the participants nor the data assessors were blinded to the treatment arms.

## Results

### Baseline Characteristics

**Figure 1:**
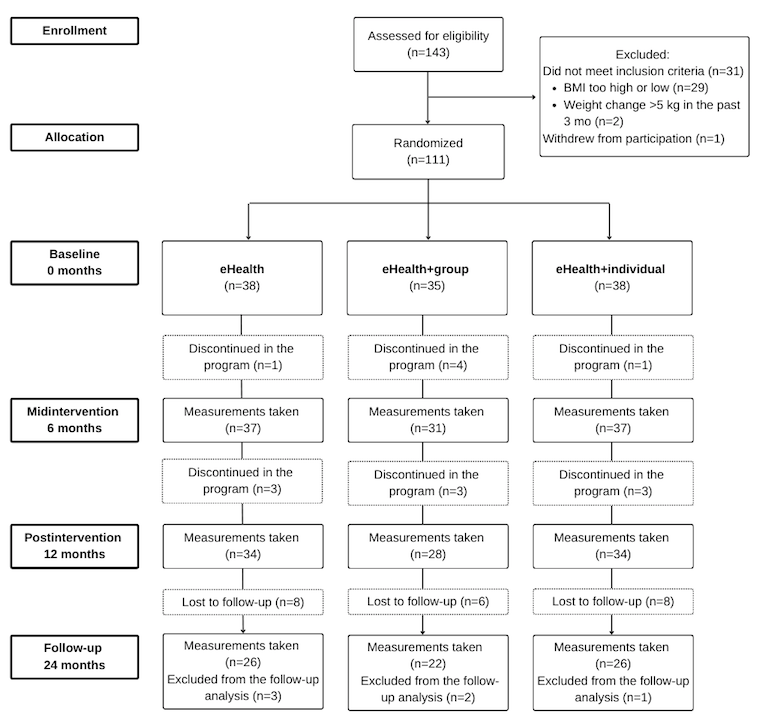
The flow of participants through the 3 treatment arms (eHealth, eHealth+group, and eHealth+individual) of the randomized controlled trial, with 6 participants’ 24-month data excluded from the analysis due to obesity medication use. All available data were included in the overall analysis.

Of the 143 people assessed for eligibility, 111 were randomized into research groups (Figure 1). Most participants were female (n=92, 83%) and worked as experts (n=46, 41%) or specialized experts (n=34, 31%; Table 1). More than half were either former or current smokers (n=59, 53%). Of the 111 participants, 73 (66%) had metabolic syndrome, 23 (21%) had mental health problems, 21 (19%) had osteoarthritis, 7 (16%) had type 2 diabetes, and 15 (14%) had sleep apnea. The mean age was 50 (SD 8.9) years, and the mean BMI was 34.2 (SD 2.9) kg/m^2^. Baseline characteristics did not differ between the groups except for triglycerides (*P*=.006), which showed statistically significant elevation in the eHealth arm compared to the other two treatment arms.

### Retention

The retention rate was 95% at the 6-month mark, 86% at the 12-month mark, and 61% at the 24-month mark of the intervention (Figure 1). The participants completed, on average, 58.6% (SD 32.7%) of the eHealth program. There were no differences in retention or progression between the treatment arms.

### Intervention Effects

We did not observe any significant between-group differences in changes in the primary outcomes of weight and categorized weight (*P*=.76 and *P*=.99, respectively; Table 2, and Tables S4 and S5 in Multimedia Appendix 1). However, we did discover an interaction between the time points and treatment arms for HDL (*P*=.03) and triglycerides (*P*=.002).

**Table 2:** Estimated mean changes (SE) in anthropometric, body composition, laboratory, and blood pressure measurements from baseline to 6, 12, and 24 months across treatment arms and all participants (N=111), analyzed using generalized estimating equations. The overall results are presented in the title row, while differences between treatment arms are shown in the corresponding rows.

Variables				0-6 months		0-12 months		0-24 months		Overall change across 0, 6, 12, and 24 months^a^		
				Mean change (SE)		Mean change (SE)		Mean change (SE)		Wald chi-square		*P* values
**Weight metrics**												
	**Weight (kg)**		–1.25 (0.35)		–1.4 (0.44)		–0.7 (0.61)		13.69		.003	
		eHealth	–0.83 (0.7)		–0.8 (0.81)		–1.25 (1.18)		3.36		.76	
		eHealth+group	–1.43 (0.7)		–1.75 (0.87)		–0.18 (0.99)		3.36		.76	
		eHealth+individual	–1.5 (0.39)		–1.69 (0.62)		–1.06 (1.06)		3.36		.76	
	**BMI (kg/m^2^)**		–0.44 (0.13)		–0.49 (0.15)		–0.29 (0.22)		13.09		.004	
		eHealth	–0.23 (0.25)		–0.24 (0.26)		–0.47 (0.42)		3.84		.70	
		eHealth+group	–0.55 (0.25)		–0.65 (0.31)		–0.11 (0.36)		3.84		.70	
		eHealth+individual	–0.54 (0.77)		–0.62 (0.23)		–0.41 (0.39)		3.84		.70	
**Body composition**												
	**Waist circumference (cm)**		–2.34 (0.35)		–2.65 (0.42)		–2.74 (0.54)		53.69		.001	
		eHealth	–2.2 (0.59)		–2.18 (0.79)		–4.07 (0.94)		6.32		.39	
		eHealth+group	–2.2 (0.73)		–2.2 (0.73)		–2.84 (0.67)		6.32		.39	
		eHealth+individual	–2.48 (0.50)		–2.93 (0.66)		–3.12 (0.85)		6.32		.39	
	**Visceral fat (cm^2^)**		–6.22 (3.54)		–5.82 (3.95)		–2.45 (4.01)		17.68		<.001	
		eHealth	–5.13 (6.23)		–3.54 (6.5)		–3.77 (6.84)		5.33		.50	
		eHealth+group	–5.48 (5.42)		–6.92 (6.57)		0.89 (6.6)		5.33		.50	
		eHealth+individual	–7.98 (6.5)		–7.23 (7.35)		–5.44 (7.29)		5.33		.50	
	**Fat percentage**		–0.75 (0.59)		–0.9 (0.64)		–0.29 (0.6)		18.2		<.001	
		eHealth	–0.77 (1.07)		–0.64 (1.08)		–0.46 (1.1)		5.28		.51	
		eHealth+group	–0.46 (0.94)		–0.86 (1)		–0.08 (0.92)		5.28		.51	
		eHealth+individual	–1 (1.05)		–1.21 (1.19)		0.62 (1.11)		5.28		.51	
	**Muscle percentage**		0.4 (0.11)		0.46 (0.14)		0.32 (0.25)		13.98		.003	
		eHealth	0.47 (0.22)		0.37 (0.23)		0.13 (0.29)		4.2		.65	
		eHealth+group	0.22 (0.2)		0.45 (0.27)		0.78 (0.68)		4.2		.65	
		eHealth+individual	0.47 (0.16)		0.58 (0.22)		0.28 (0.3)		4.2		.65	
**Laboratory measurements**												
	**Hemoglobin A_1c_(mmol/L)**		–0.38 (0.32)		0.62 (0.34)		0.07 (0.4)		10.89		.01	
		eHealth	–0.39 (0.56)		0.65 (0.55)		0.19 (0.61)		5.49		.48	
		eHealth+group	0.3 (0.59)		0.42 (0.72)		0.22 (0.76)		5.49		.48	
		eHealth+individual	–0.97 (0.5)		0.72 (0.51)		0.03 (0.74)		5.49		.48	
	**Glucose (mmol/L)**		–0.34 (0.06)		–0.35 (0.06)		–0.24 (0.06)		45.59		<.001	
		eHealth	–0.5 (0.1)		–0.37 (0.1)		–0.18 (0.14)		9.49		.15	
		eHealth+group	–0.21 (0.11)		–0.39 (0.12)		–0.35 (0.11)		9.49		.15	
		eHealth+individual	–0.3 (0.08)		–0.3 (0.09)		–0.21 (0.08)		9.49		.15	
	**Cholesterol (mmol/L)**		–0.11 (0.08)		–0.13 (0.08)		–0.11 (0.11)		2.89		.41	
		eHealth	–0.24 (0.11)		–0.24 (0.13)		–0.22 (0.17)		6.8		.34	
		eHealth+group	–0.01 (0.19)		–0.14 (0.16)		–0.23 (0.22)		6.8		.34	
		eHealth+individual	–0.07 (0.09)		–0.008 (0.1)		0.13 (0.16)		6.8		.34	
	**High-density lipoprotein (mmol/L)**		0.05 (0.02)		0.07 (0.02)		0.11 (0.02)		29.58		<.001	
		eHealth	0.04 (0.07)		0.05 (0.08)		0.17 (0.07)		14.23		.03	
		eHealth+group	0.06 (0.04)		0.11 (0.04)		0.09 (0.04)		14.23		.03	
		eHealth+individual	–0.01 (0.02)		0 (0.03)		0.13 (0.04)		14.23		.03	
	**Low-density lipoprotein (mmol/L)**		–0.04 (0.07)		–0.13 (0.07)		–0.11 (0.1)		4		.26	
		eHealth	–0.11 (0.10)		–0.14 (0.12)		–0.14 (0.15)		6.16		.41	
		eHealth+group	0.01 (0.17)		–0.2 (0.16)		–0.27 (0.2)		6.16		.41	
		eHealth+individual	–0.02 (0.08)		–0.06 (0.1)		0.09 (0.15)		6.16		.41	
	**Triglycerides (mmol/L)**		–0.21 (0.07)		–0.12 (0.07)		–0.21 (0.07)		13.34		.004	
		eHealth	–0.47 (0.12)		–0.42 (0.14)		–0.46 (0.11)		20.87		.002	
		eHealth+group	–0.09 (0.15)		–0.06 (0.13)		–0.18 (0.13)		20.87		.002	
		eHealth+individual	–0.05 (0.07)		0.13 (0.06)		0.02 (0.1)		20.87		.002	
	**Alanine aminotransferase (U/L)**		–4.6 (1.88)		–3.77 (2.12)		–3.08 (2.2)		6.57		.09	
		eHealth	–6.04 (4.41)		–5.35 (4.81)		–4.16 (5.06)		2.37		.88	
		eHealth+group	–3.13 (2.45)		–1.01 (2.87)		–3.59 (2.25)		2.37		.88	
		eHealth+individual	–4.69 (2.18)		–4.74 (2.66)		–1.29 (3.39)		2.37		.88	
	**High-sensitivity C-reactive protein (mg/L)**		0.28 (0.19)		0.16 (0.27)		0.83 (0.3)		9.05		.03	
		eHealth	0.48 (0.39)		–0.02 (0.35)		1.69 (0.63)		8.39		.21	
		eHealth+group	0.34 (0.29)		0.4 (0.56)		0.9 (0.53)		8.39		.21	
		eHealth+individual	0 (0.28)		0.09 (0.49)		0.06 (0.39)		8.39		.21	
**BP^b^**												
	**Systolic BP (mm Hg)**		–2.88 (1.12)		–2.22 (1.32)		–0.96 (1.7)		7.67		.053	
		eHealth	–4.15 (2.22)		–3.82 (2.35)		–1.09 (3.06)		3.81		.7	
		eHealth+group	–3.27 (1.78)		–1.36 (2.61)		0.97 (4.03)		3.81		.7	
		eHealth+individual	–1.24 (1.68)		–1.3 (1.91)		–2.13 (1.88)		3.81		.7	
	**Diastolic BP (mm Hg)**		–2.37 (0.67)		–2.33 (0.65)		–1.15 (0.99)		17.64		<.001	
		eHealth	–3.18 (1.25)		–3 (1.22)		–2.4 (1.81)		5.13		.53	
		eHealth+group	–2.34 (0.93)		–1.38 (1.01)		1.62 (1.95)		5.13		.53	
		eHealth+individual	–1.52 (1.17)		–2.38 (1.08)		–1.68 (1.23)		5.13		.53	
	**Pulse (beats per minute)**		–0.44 (0.7)		0.06 (0.75)		0.89 (0.79)		2.23		.53	
		eHealth	–0.35 (1.14)		1.11 (1.25)		1.53 (1.21)		2.77		.84	
		eHealth+group	0.06 (1.2)		0.04 (1.18)		2.31 (1.45)		2.77		.84	
		eHealth+individual	–0.71 (1.3)		–0.59 (1.41)		–0.48 (1.46)		2.77		.84	

^a^Degrees of freedom=6 for the between-group analysis and degrees of freedom=3 for the single-group analysis, including all participants.

^b^BP: blood pressure.

Considering HDL, further analysis showed a greater increase in the eHealth arm compared to the eHealth+individual arm between 0 and 6 months (*P*=.09, *d*=0.35) and 0-12 months (*P*=.03, *d*=0.28). The interaction between the time points and treatment arms was explained by sex (*P*=.002), cholesterol medication use (*P*<.001), and changes in cholesterol medication (*P*<.001). When participants who were using cholesterol medication at baseline or had changes in their medication were excluded, the interaction between the treatment arm and time point was insignificant (*P*=.17). This suggests that the difference in HDL between the treatment arms was likely due to variations in medication use.

For triglycerides, the eHealth arm demonstrated a greater decrease than the eHealth+individual arm at all time points relative to baseline (0-6 months *P*=.03, *d*=0.41; 0-12 months *P*<.001, *d*=0.53; 0-24 months *P*=.002, *d*=0.47; Table 2). However, the eHealth arm had higher triglyceride levels at baseline compared to the other groups (Table 1).

To study the overall effect of the intervention, the 3 groups were considered as a single eHealth group and combined for further analysis, as reported below.

### Overall Impact

#### Weight

**Figure 2:**
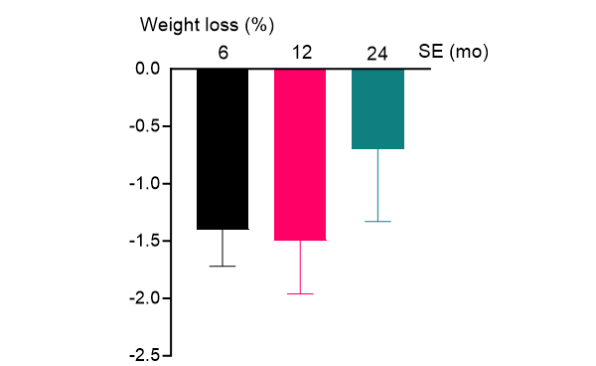
Weight loss (%) and SE midintervention (6 months), postintervention (12 months), and at follow-up (24 months) compared to baseline (0 months) displaying estimated values used by the generalized estimating equations.

At the 6- and 12-month mark of the intervention, participants experienced an estimated mean weight loss of 1.3 kg (1.4%, *P*<.001) and 1.4 kg (1.5%, *P*=.002), respectively, compared to baseline (ITT analysis presented in Table 2 and Figure 2, and completer analysis in Table S6 in Multimedia Appendix 1). Overall, weight showed a significant decrease over the 24-month study period (*P*=.003) when analyzed across all time points (0, 6, 12, and 24 months).

**Figure 3:**
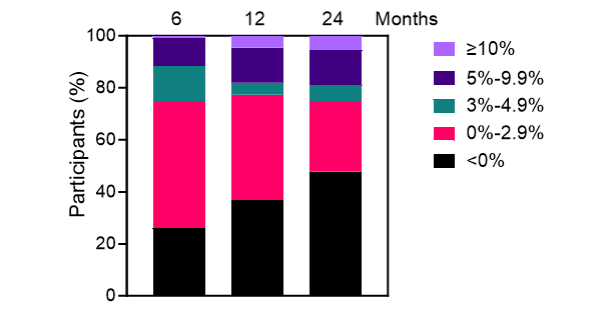
Categorized weight loss (%) midintervention (6 months), postintervention (12 months), and at follow-up (24 months; N=111) with last observation carried forward.

Using the entire sample of 111 participants and applying the last observation carried forward principle, categorical analysis at the 6-month time point revealed that 15 (14%) participants had lost 3%-4.9% of their baseline body weight, 12 (11%) participants had lost 5%-9.9%, and 1 (1%) participant had lost ≥10% (Figure 3). At 12 months, 5 (5%) participants had lost 3%-4.9% of their baseline body weight, 15 (14%) participants lost 5.2%-9.9%, and 5 (5%) participants lost ≥10%. Similarly, at the 24-month follow-up, the respective proportions in each category were 7 (6%) participants, 15 (14%) participants, and 6 (5%) participants. Overall, 20 of 111 (18%) participants achieved a clinically relevant weight loss of ≥5% of their initial weight at the 12-month mark, increasing to 21 (19%) participants at the 24-month mark.

The weight changes were correlated between different measurement points. We observed strong correlations between the 0-6–month weight change and 0-12–month weight change (*r*=0.64, *P*<.001), and between the 0-12–month weight change and 0-24–month weight change (*r*=0.66, *P*<.001). The correlation was moderate between the 0-6–month weight change and 0-24–month weight change (*r*=0.324, *P*=.008).

#### Waist Circumference and Body Composition

The estimated mean decrease in waist circumference was 2.4 cm (SD 0.35 cm; *P*<.001) and 2.6 cm (SD 0.42 cm; *P*<.001) at the 6- and 12-month marks of the intervention, respectively (Table 2, Table S6 in Multimedia Appendix 1). At follow-up, the waist circumference continued to decrease, showing a 2.8 cm mean decrease from 0 to 24 months (SD 0.54 cm; *P*<.001).

The estimated mean decrease in visceral fat was 6.2 cm^2^ (SD 3.54 cm^2^; *P*<.001) and 5.8 cm^2^ (SD 3.95 cm^2^; *P*=.003) at the 6- and 12-month marks of the intervention, respectively (Table 2, Table S6 in Multimedia Appendix 1). Fat percentage decreased by 0.8% (SD 0.59%, *P*<.001) and 0.9% (SD 0.64%, *P*<.001) at the 6- and 12-month marks. Muscle percentage increased by 0.4% (0.11%, *P*<.001) and 0.5% (SD 0.14%, *P*<.001) at the 6- and 12-month marks.

The covariate analysis showed a significant interaction for baseline weight with changes in waist circumference, visceral fat, fat percentage, and muscle percentage (Table S7 in Multimedia Appendix 1). Post hoc analysis revealed that participants with lower baseline weight had a greater reduction in waist circumference, visceral fat, fat percentage, and a greater increase in muscle percentage. There was also a significant covariate interaction between age and waist circumference, with older participants having a greater reduction in waist circumference.

#### Laboratory Measurements and Blood Pressure

The estimated fasting glucose decrease was 0.3 mmol/L at 6 months (*P*<.001), 0.4 at 12 months (*P*<.001), and 0.2 (*P*<.001) at 24 months (Table 2 and Table S6 in Multimedia Appendix 1). There was a significant interaction between diabetes medicine usage and glucose level change, and changes in diabetes medication usage and glucose level change, with greater glucose level reductions observed for participants who had existing medication or who were prescribed new medication to manage blood glucose levels (Table S6 in Multimedia Appendix 1). However, the results of the main analysis remained after excluding these participants (n=9) from the analysis (0-12–month *P*<.001; 0-24–month *P*=.002).

There was a statistically significant overall change in HbA_1c_ (*P*=.01), with the estimated mean value increase of 0.62 mmol/L at 12 months and 0.07 at 24 months. However, these changes were not significant compared to the baseline. Changes in HbA_1c_ levels were significantly associated with changes in diabetes medication (*P*<.001), with participants who reduced their diabetes medication (n=12) during the intervention showing a greater reduction in HbA_1c_ levels than those who increased or started new medications or who made no changes. However, the main result remained unchanged after excluding the participants with increased or new diabetes medication (n=24).

The HDL increase was 0.1 mmol/L at the 6-month (*P*=.007), 12-month (*P*<.001), and 24-month (*P*<.001) marks of the intervention (Table 2 and Table S6 in Multimedia Appendix 1). The triglycerides decrease was 0.2 mmol at both 6-months (*P*=.002) and 24-months (*P*=.004). There was a statistically significant interaction between the changes in cholesterol medicine with HDL (*P*<.001) and triglycerides (*P*<.001), but the result of the main analysis remained after excluding these participants (n=2) from the analysis (*P*<.001 and *P*=.01, respectively; Table S7 in Multimedia Appendix 1).

The hs-CRP increased by 0.9 mg/L from baseline to 24-month follow-up (*P*=.004) when outliers with severe elevation of >30 mg/L were excluded (n=4; Table 2 and Table S6 in Multimedia Appendix 1). The result of the pairwise comparison remained significant (*P*=.01) when lowering the outlier threshold to 10 mg/L (marked elevation). Diastolic blood pressure decrease was 2.4 mm Hg at the 6-month (*P*<.001) and 2.3 mm Hg at the 12-month (*P*<.001) marks of the intervention. Covariate analysis flagged a significant interaction between changes in blood pressure medication and diastolic blood pressure (*P*<.001; Table S7 in Multimedia Appendix 1). Nevertheless, the results persisted (*P*=.002) even after excluding participants (n=6) who had medication increase or initiation.

Metabolic syndrome prevalence was 66% at baseline (Table 1) and 60% in both 12-month postintervention and 24-month follow-up, indicating a change of -9%. These changes were not statistically significant.

## Discussion

### Principal Findings

This study examined whether adding minimal individual or group-based face-to-face support to a 12-month coach-assisted eHealth intervention enhances outcomes by strengthening connections with coaches or leveraging peer support. Contrary to our hypothesis, no significant differences in primary outcomes were found between the groups, indicating that 3 live sessions—whether individual or group-based—did not provide additional benefits over the eHealth protocol alone. Interestingly, the eHealth-only group showed greater improvements in HDL cholesterol and triglycerides compared to the individual session group. However, post hoc analyses attributed these differences to cholesterol medication use and higher baseline triglyceride levels rather than the intervention itself. Thus, incorporating infrequent live sessions into the eHealth protocol does not appear to offer additional advantages.

In the entire study population, the intervention resulted in a mean weight loss of 1.5% from baseline to 12-month postintervention and 0.7% from baseline to 24-month follow-up. When considering individual weight change, the results are polarized: the received support promoted weight loss for some participants, while others did not respond. The weight changes were notably correlated between different measurement points, suggesting the individual weight change to be rather consistent.

Throughout the intervention and follow-up, there was a progressive decrease in mean waist circumference, along with improvements in the body composition variables at the 12-month mark.

Additionally, there was a slight enhancement in HDL cholesterol levels at postintervention and follow-up, suggesting positive effects on lipid metabolism. Although we observed a decrease in the fasting glucose levels during the intervention and follow-up, similar results were not observed in HbA_1c_, a marker measuring the average blood glucose levels over the past few months. This indicates that the observed improvements in glucose levels might not be consistent or lasting enough to affect overall glycemic control.

Unexpectedly, the hs-CRP increased significantly by the 24-month follow-up, suggesting elevated inflammation not attributable to outliers but potentially reflecting mild infections or systemic inflammation [44]. We do not attribute the increase in the hs-CRP to heightened metabolic disturbances, as we noted a slight, though statistically insignificant, improvement in the prevalence of metabolic syndrome.

### Comparison With Prior Work

There are several potential explanations for the lack of intergroup differences. It is possible that only 3 sessions in the hybrid conditions do not yield a sufficient difference compared to the coach-assisted eHealth intervention, and by increasing the number of sessions in the hybrid intervention, differences between the groups could be observed. In Teeriniemi et al [29], participants with a BMI over 30 kg/m^2^ lost an average of 4.4% of their weight in 12 months when they received a hybrid intervention combining eHealth with 8 group meetings based on cognitive behavioral therapy, compared to just 1.6% weight loss in the eHealth-only arm. Therefore, a more intensively enhanced hybrid approach may be needed to further improve somatic outcomes.

Notably, the intervention took place during the COVID-19 era, leading to all group and individual meetings being conducted remotely. As seen in previous research on therapeutic relationships [45], this shift could have hindered the alliance between the participants and the coach. Particularly, the unplanned move to remote group meetings in the eHealth+group treatment arm may have affected the group’s cohesion and perceived peer support [46], thereby impacting effectiveness and attenuating differences between the treatment arms.

Overall, it is likely that differences in weight change between the 3 study arms are attenuated by the lack of power resulting from smaller-than-expected changes in weight, with high variability. The total weight loss is modest compared to the 2019 meta-analysis by Lawlor [12], reporting a mean absolute weight change of 5.5 kg in third-wave cognitive behavior therapies. Furthermore, the weight loss outcomes observed in our study fall short in comparison to the results reported from previous implementations of the HWC eHealth program, which achieved a 4.6% weight loss during the 12-month intervention [47]. Additionally, a “review of reviews” by Kupila et al [23] reported mean differences in weight loss results ranging from –0.12 (95% CI –0.64 to 0.41) kg in eHealth interventions compared with face-to-face care to –4.32 (–5.08 to –3.57) kg in eHealth interventions compared with no care. However, our results align with the 12-month weight loss of –2.08 kg reported by Mueller et al [33] for another ACT-based hybrid intervention (eHealth+phone support) conducted during the COVID-19 pandemic.

Several factors could contribute to the lower weight loss observed in our study. First, our study used a more weight-neutral coaching style than the one in Kupila et al [47], which may have impacted its effectiveness in promoting weight loss compared to the earlier version. It needs to be noted that a newer version of the HWC is already in clinical use. Therefore, the results of our study do not demonstrate the current effectiveness of the HWC.

Second, our study involved a different population characterized by lower BMI compared to the study of Kupila et al [47]. In their study, the greatest weight loss was observed among individuals with a baseline BMI ≥40 kg/m², a subgroup that was not included in our study. Yet, in Kupila et al [47], the mean weight loss for those with a baseline BMI <40 kg/m² was 3.2%—more than the weight change observed here.

Third, the influence of the COVID-19 pandemic coinciding with the 2021-2022 intervention cannot be overlooked, as research suggests that the pandemic led to disruptions in lifestyle behaviors that could affect weight management. The mean weight gain during the pandemic was higher [48] than average [49], indicating difficulty in weight maintenance. These effects are evident in the Finnish population: between 2017 and 2023, the prevalence of obesity increased 3%-points in men and 4%-points in women [50]. In contrast, the prevalence of obesity had remained unchanged between 2012 and 2017 [51]. In addition, an increase in food consumption and sedentary activity was observed during the pandemic [52]—changes that were potentially intensified in this study population, most of whom worked in expert positions and shifted to remote work during the pandemic.

Lastly, our study involved a different population characterized by significant occupational burden from mentally consuming expert work. This occupational burden may have posed challenges to weight management despite participation in the intervention, especially with the combination of the eHealth program and remote knowledge work. This is per the earlier results showing that early weight loss predicts 1-year weight loss in web-based programs [53] and shows that the early weight loss could even predict long-term weight maintenance.

In the secondary analysis with the overall sample, long-term improvements in waist circumference and lipid metabolism are consistent with findings from previous nondiet studies [54-57]. Additionally, the increase in muscle mass contrasts with previous findings of skeletal muscle mass loss in weight loss studies [58]. These changes are likely attributed to our intervention’s focus on guiding participants toward making healthy dietary choices and increasing physical activity without imposing calorie restrictions. However, our findings of no improvement in long-term glucose metabolism contrast with previous studies showing that lifestyle weight loss interventions reduce the risk of elevated fasting glucose levels and HbA_1c_ [59-61], suggesting that a more prominent weight loss may be necessary to achieve sustained improvements in glycemic control.

### Strengths and Limitations

This study presents both strengths and limitations. A key strength is the long follow-up period, which allowed for the investigation of the treatment’s long-term effects—rare in typical ACT-based interventions. Additionally, the comprehensive array of outcome measures, including body composition and laboratory tests alongside traditional metrics such as weight and waist circumference, offers a holistic understanding of the intervention’s effects. This study also ensured accuracy and reliability by conducting all measurements and laboratory tests on-site despite the onset of COVID-19. Additionally, the emergence of new weight loss medications was addressed by screening and excluding participants using weight loss medications, requesting that participants refrain from starting new weight loss medications during the intervention, regularly collecting information on any changes in medication, and excluding from the analysis those who began using them during the follow-up period.

However, some limitations impact especially the interpretation of the overall study effects. First, the participant group was homogeneous—all employed and primarily middle-aged women in expert professions—making generalization to other populations challenging. Second, the lack of blinding and participants’ awareness of their treatment arms before randomization could have affected motivation, particularly for those not assigned to their preferred group. Third—affecting interpretation of the overall results—the absence of an inactive control group prevents comparison against a baseline, making it difficult to isolate the treatment effects from external influences or placebo effects and thus establish causality. These limitations, arising from the integration of this study within routine occupational health care services and the need for ethical service provision to willing participants, highlight important considerations for future research aiming to balance real-world applicability with rigorous study design.

### Conclusions

Our study found no differences in weight or other somatic health variables between the eHealth arm and intervention combining eHealth with minimal group or individual enhancement. Moving forward, further research is needed to determine if there is a threshold where face-to-face meetings provide additional benefits in hybrid interventions. Moreover, there is a need to explore for whom and under what conditions eHealth and hybrid models may be most effective.

## Appendix

Multimedia Appendix 1 Supplemental tables.

Multimedia Appendix 2 CONSORT eHEALTH checklist (V 1.6.1).

## Abbreviations

%-point: percentage point
ACT: acceptance and commitment therapy
GEE: generalized estimating equation
HbA1c: hemoglobin A1c
HDL: high-density lipoprotein
hs-CRP: high-sensitivity C-reactive protein
HWC: Healthy Weight Coaching
ITT: intention-to-treat analysis

## Data Availability

The datasets generated or analyzed during this study are available from the corresponding author on reasonable request.
