# Supplementary material for: Coach-Assisted eHealth With Group or Individual Support for Employees With Obesity: Randomized Controlled Trial on Weight, Body Composition, and Health Metrics
Source: J Med Internet Res. 2025 Mar 12;27:e60436. doi: 10.2196/60436 (PMC11947631; doi:10.2196/60436)
Supplement: Multimedia Appendix 1 [file jmir_v27i1e60436_app1.docx]

| **Table S1.** Central themes of the Healthy Weight Coaching program, incorporating behavioral weight management and acceptance and commitment therapy (ACT). | | |
| --- | --- | --- |
|  | **Theme** | **E.g. subthemes and exercises** |
| **Behavioral weight management** | Eating habits | Meal rhythm diary, picture food diary  Plan for adding vegetables, the role of sugar in the diet  Hunger scale |
|  | Physical activity | Choose tips for increasing everyday movement  Neighborhood walk  Mini-exercises |
|  | Sleep | Factors affecting sleep and examining one's own sleep habits  The effect of alcohol on sleep  Sleep-promoting movement |
|  | Stress management | Stress management techniques and personal ways to regulate stress  Give yourself relaxation moments  The effect of stress on bodily functions |
| **ACT processes** | Values | Things important to you |
|  | Committed action | Everyday goals and actions  Actions that fit your life situation and values |
|  | Contact with the present moment | Following your breath  Body scan  Mindful eating |
|  | Acceptance | Self-compassion and acceptance in the present moment (thoughts, feelings, bodily sensations, pace of change) |
|  | Defusion | Important actions even with unpleasant emotions  Exposure to temptations  Internal dialogue and thoughts sabotaging change |
|  | Self as context | Lifestyle changes through self-esteem  Weight and body image  My strengths |

**Table S2.** Themes of the group meetings in the eHealth+group treatment arm.

| **Meeting** | **Theme** | **Psychoeducation** | **Exercise** | **Pair/group discussion** |
| --- | --- | --- | --- | --- |
| 1 | Flexibility in weight loss | The paradox of weight loss, flexible restraint in eating and exercising | “Don’t think about the jelly-filled donuts” | Lessons learned from previous weight loss attempts  Where could I introduce more flexibility? Where do I feel flexibility is already achievable? |
| 2 | Stress regulation and mindfulness | The impact of stress-management in weight loss, mindfulness, consistency and moderation | Mindful breathing | What restores you and how is it part of your daily life? Experiences with mindfulness? |
| 3 | Self-compassion and acceptance | Acceptance and self-compassion in lifestyle change. changing body | Difficult moment. The observing self | What are you grateful for in your body today?  Realizations over the intervention, what would you like to carry forward into the future? |

| **Table S3.** Changes in medication usage. | | | |
| --- | --- | --- | --- |
|  | **Decreased** | **Increased** | **Initiated** |
| **Diabetes medication** | 0 | 0 | 2 |
| **Blood pressure medication** | 3 | 2 | 4 |
| **Cholesterol medication** | 0 | 0 | 2 |

**Table S4.** Completers’ weight change in treatment arms categorized by the magnitude of weight loss percentage, analyzed by Pearson chi-square.

|  | **0–12 months** | | | | | **0–24 months** | | | | |
| --- | --- | --- | --- | --- | --- | --- | --- | --- | --- | --- |
|  | **< 0 %** | **-0.1 to -4.9 %** | **-5 to 9.9 %** | **> -9.9 %** | **N** | **< 0 %** | **-0.1 to -4.9 %** | **-5 to 9.9 %** | **> -9.9 %** | **N** |
| **eHealth** | 17 (50%) | 12 (35%) | 4 (12%) | 1 (3%) | 34 | 12 (52%) | 5 (22%) | 4 (17%) | 2 (9%) | 23 |
| **eHealth+Group** | 8 (29%) | 13 (46%) | 6 (21%) | 1 (4%) | 28 | 10 (50%) | 6 (30%) | 3 (15%) | 1 (5%) | 20 |
| **eHealth+Individual** | 15 (44%) | 12 (35%) | 4 (12%) | 3 (9%) | 34 | 12 (48%) | 6 (24%) | 4 (16%) | 3 (12%) | 25 |
| **Total** | 40 (42%) | 37 (39%) | 14 (15%) | 5 (5%) | 96 | 34 (50%) | 17 (25%) | 11 (16%) | 6 (9%) | 68 |
|  | Between-group *P*=0.543 | | | | | Between-group *P*=0.985 | | | | |

**Table S5.** Completers' mean (SE) changes from baseline to 6, 12, and 24 months by treatment arm (eHealth [n=23], eHealth+group [n=20], eHealth+individual [n=25]) – statistical analysis conducted using the generalized estimating equations with completer data.

|  | **0 to 6 mo change** | | | **0 to 12 mo change** | | | **0 to 24 mo change** | | |  |
| --- | --- | --- | --- | --- | --- | --- | --- | --- | --- | --- |
|  | **eHealth**  **(N=37)** | **eHealth+**  **Group (N=31)** | **eHealth+**  **Indiv.**  **(N=37)** | **eHealth**  **(N=34)** | **eHealth+**  **Group (N=28)** | **eHealth+**  **Indiv.**  **(N=34)** | **eHealth**  **(N=23)** | **eHealth+**  **Group (N=20)** | **eHealth+**  **Indiv.**  **(N=25)** | ***P*^^^** |
| **Antropometric measurements** |  |  |  |  |  |  |  |  |  |  |
| Weight (kg) | -1.94 (0.7) | -2.45 (0.8) | -1.95 (0.5) | -1.96 (1.0) | -2.45 (1.0) | -2.28 (0.8) | -1.17 (1.2) | -0.64 (1.0) | -1.23 (1.0) | .915 |
| BMI (kg/m^2^) | -0.56 (0.2) | -0.84 (0.3) | -0.74 (0.2) | -0.62 (0.3) | -0.87 (0.3) | -0.86 (0.4) | -0.39 (0.4) | -0.23 (0.4) | -0.49 (0.4) | .902 |
| Waist circumference (cm) | -2.97 (0.7) | -2.92 (0.7) | -2.68 (0.6) | -3.54 (0.9) | -2.80 (0.8) | -3.26 (0.8) | -3.89 (0.9) | -2.00 (1.2) | -3.20 (0.9) | .880 |
| **Body composition** |  |  |  |  |  |  |  |  |  |  |
| Visceral fat (cm^2^) | -10.33 (3.0) | -9.54 (3.4) | -9.49 (2.3) | -7.94 (3.9) | -9.58 (4.4) | -8.70 (3.4) | -4.77 (4.5) | -0.72 (3.5) | -6.24 (4.2) | .518 |
| Fat percentage (%) | -1.28 (0.4) | -0.94 (0.4) | -1.28 (0.4) | -1.18 (0.4) | -1.18 (0.5) | -1.44 (0.5) | -0.70 (0.5) | -0.31 (0.4) | -0.75 (0.6) | .916 |
| Muscle percentage (%) | 0.83 (0.2) | 0.47 (0.2) | 0.62 (0.2) | 0.62 (0.2) | 0.65 (0.3) | 0.69 (0.3) | 0.31 (0.3) | 0.86 (0.7) | 0.34 (1.3) | .739 |
| **Laboratory measurements** |  |  |  |  |  |  |  |  |  |  |
| HbA1c (mmol/mol) | -1.41 (0.7) | 0.40 (0.6) | -0.77 (0.6) | 0.30 (0.7) | 1.45 (0.9) | 0.99 (0.7) | -0.83 (0.5) | 0.67 (0.8) | 0.20 (0.8) | .438 |
| Glucose (mmol/l) | -0.56 (0.2) | -0.26 (0.1) | -0.30 (0.1) | -0.45 (0.1) | -0.35 (0.1) | 0.30 (0.1) | -0.29 (0.1) | -2.30 (0.1) | -0.20 (0.1) | .710 |
| Cholesterol (mmol/l) | -0.40 (0.2) | -0.07 (0.3) | -0.01 (0.1) | -0.40 (0.2) | -0.08 (0.2) | 0.05 (0.1) | -0.45 (0.2) | -0.20 (0.3) | 0.25 (0.1) | .122 |
| High-Density Lipoprotein (mmol/l) | 0.14 (0.0) | 0.09 (0.0) | -0.02 (0.0) | 0.14 (0.0) | 0.15 (0.0) | 0.02 (0.0) | 0.15 (0.0) | 0.12 (0.0) | 0.09 (0.0) | .032 |
| Low-Density Lipoprotein (mmol/l) | -0.24 (0.1) | -0.05 (0.3) | 0.01 (0.1) | -0.27 (0.2) | -0.14 (0.2) | -0.02 (0.1) | -0.35 (0.2) | -0.28 (0.3) | 0.17 (0.1) | .208 |
| Triglycerides (mmol/l) | -0.55 (0.1) | -0.07 (0.2) | -0.01 (0.1) | -0.56 (0.2) | -0.18 (0.1) | 0.01 (0.1) | -0.49 (0.1) | -0.09 (0.1) | 0.05 (0.1) | <.001 |
| Alanine aminotransferase (U/l) | -8.82 (6.4) | -4.35 (3.6) | -4.80 (3.1) | -7.91 (7.0) | -3.20 (4.1) | -5.04 (3.4) | -5.57 (6.2) | -4.85 (2.3) | -2.21 (3.8) | .984 |
| High-sensitivity C-reactive protein (mg/l) | 0.29 (0.3) | 0.35 (0.4) | 0.03 (0.4) | 0.10 (0.24) | 0.42 (0.8) | 0.42 (0.6) | 1.34 (0.6) | 0.42 (0.8) | 0.28 (0.4) | .422 |
| **Blood pressure** |  |  |  |  |  |  |  |  |  |  |
| Systolic (mmHg) | -6.74 (3.3) | -0.88 (1.9) | -1.47 (1.9) | -5.65 (3.3) | 0.25 (3.3) | 0.84 (1.8) | -3.20 (3.7) | 2.58 (4.2) | -2.28 (1.6) | .399 |
| Diastolic (mmHg) | -5.34 (1.7) | -2.10 (1.3) | -2.15 (1.4) | -4.37 (1.7) | -0.83 (1.1) | -2.96 (1.1) | -4.54 (2.1) | 2.08 (2.2) | -2.52 (1.2) | .208 |
| Pulse (BPM) | -2.97 (1.9) | -0.60 (1.1) | -1.00 (1.8) | -1.09 (1.8) | 0.38 (1.5) | -1.48 (1.6) | -0.41 (1.6) | 2.90 (1.3) | -0.74 (1.5) | .509 |

^^^*P* value indicating significance of the between-group change across the four measurement points (0-6-12-24 mo)

**Table S6.** Completers’ mean (SE) changes in anthropometric, body composition, laboratory, and blood pressure measurements from baseline to 6, 12, and 24 months in all study participants – statistical analyses performed using generalized estimating equations with completer data.

|  | 0 to 6 mo change | |  | 0 to 12 mo change | |  | 0 to 24 mo change | |  | Overall change 0-6-12-24 mo | |
| --- | --- | --- | --- | --- | --- | --- | --- | --- | --- | --- | --- |
|  |  |  |  |  |  |  |  |  |  |  |  |
|  | Total (N=105) | *P*^^^ | d^⊥^ | Total (N=96) | *P*^^^ | d^⊥^ | Total (N=68) | *P*^^^ | d^⊥^ | Wald-chi square (df=3) | *P*^^^ |
| **Antropometric measurements** | | |  |  |  |  |  |  |  |  |  |
| Weight (kg) | -1.4 (0.3) | <.001 | 0.17 | -1.4 (0.5) | .002 | 0.17 | -1.0 (0.6) | .27 | 0.18 | 19.33 | <.001 |
| Weight (%) | -1.4 (0.3) | <.001 |  | -1.5 (0.5) | .002 |  | -1.2 (0.7) | .61 |  | 19.47 | <.001 |
| BMI (kg/m^2^) | -0.4 (0.1) | <.001 | 0.17 | -0.5 (0.2) | .002 | 0.19 | -0.4 (0.2) | .25 | 0.15 | 19.40 | <.001 |
| Waist circumference (cm) | -2.3 (0.4) | <.001 | 0.28 | -2.6 (0.4) | <.001 | 0.32 | -3.0 (0.6) | <.001 | 0.43 | 54.83 | <.001 |
| **Body composition** | |  |  |  |  |  |  |  |  |  |  |
| Visceral fat (cm2) | -6.3 (1.5) | <.001 | 0.16 | -6.0 (2.0) | .002 | 0.14 | -4.1 (2.4) | .33 | 0.11 | 18.79 | <.001 |
| Fat percentage (%) | -0.8 (0.2) | <.001 | 0.12 | -0.9 (0.3) | <.001 | 0.11 | -0.6 (0.3) | .36 | 0.05 | 18.43 | <.001 |
| Muscle percentage (%) | 0.4 (0.1) | <.001 | 0.11 | 0.5 (0.1) | <.001 | 0.08 | 0.4 (0.2) | .20 | 0.05 | 13.66 | .003 |
| **Laboratory measurements** | | |  |  |  |  |  |  |  |  |  |
| HbA1c (mmol/mol) | -0.9 (0.6) | .19 | 0.18 | 0.03 (0.6) | .07 | 0.02 | -0.9 (0.9) | .68 | 0.10 | 11.81 | .008 |
| Glucose (mmol/l) | -0.4 (0.1) | <.001 | 0.46 | -0.5 (0.1) | <.001 | 0.46 | -0.4 (0.2) | <.001 | 0.29 | 46.09 | <.001 |
| Cholesterol (mmol/l) | -0.1 (0.08) | .16 | 0.20 | -0.1 (0.1) | .10 | 0.20 | -0.1 (0.1) | .33 | 0.20 | 2.86 | .41 |
| High-Density Lipoprotein (mmol/l) | 0.05 (0.02) | .009 | 0.33 | 0.1 (0.02) | <.001 | 0.33 | 0.1 (0.02) | <.001 | 0.33 | 29.51 | <.001 |
| Low-Density Lipoprotein (mmol/l) | -0.05 (0.07) | .59 | 0.11 | -0.1 (01) | .07 | 0.22 | -0.2 (0.1) | .27 | 0.22 | 4.26 | .24 |
| Triglycerides (mmol/l) | -0.2 (0.07) | .002 | 0.22 | -0.1 (0.1) | .09 | 0.11 | -0.1 (0.1) | .006 | 0.22 | 13.28 | .004 |
| Alanine aminotransferase (U/l) | -4.9 (1.9) | .01 | 0.19 | -3.8 (2.2) | .08 | 0.13 | -3.2 (2.5) | .17 | 0.10 | 6.58 | .09 |
| High-sensitivity C-reactive protein (mg/l) | 0.3 (0.2) | .13 | -0.11 | 0.1 (0.3) | .56 | -0.07 | 0.9 (0.3) | .006 | -0.31 | 7.98 | .05 |
| **Blood pressure** | |  |  |  |  |  |  |  |  |  |  |
| Systolic (mmHg) | -2.7 (1.1) | .01 | 0.24 | -2.3 (1.4) | .09 | 0.17 | -1.0 (1.9) | .51 | 0.14 | 6.86 | .08 |
| Diastolic (mmHg) | -2.3 (0.7) | <.001 | 0.30 | -2.3 (0.7) | <.001 | 0.32 | -1.8 (1.1) | .22 | 0.24 | 17.16 | <.001 |
| Pulse (BPM) | -0.2 (0.7) | .54 | 0.09 | -0.1 (0.8) | .92 | 0.06 | 1.1 (0.8) | 0.20 | 0.02 | 2.54 | .47 |

^^^ *P* value indicates the statistical significance of the change at post-intervention (pairwise comparison of 0 to 12 mo change) and follow up (pairwise comparison of 0 to 24 mo change), and the overall change during the four measurement points (0, 6, 12, 24 mo), respectively

^⊥^ d indicates Cohen’s d effect size

| **Table S7.** Interactions between measurements, time points (0, 6, 12, and 24 months), and covariates (baseline weight, age, sex, medication usage, and medication changes), analyzed for the entire study population using the general estimating equations. | | | | | | | | | | |
| --- | --- | --- | --- | --- | --- | --- | --- | --- | --- | --- |
| **Measurements** | **Baseline weight** | | **Age** | | **Sex** | | **Medication usage** | | **Medication change** | |
|  | **Wald chi-square (df=3)** | ***P*^^^** | **Wald chi-square (df=3)** | ***P* ^^^** | **Wald chi-square (df=3)** | ***P* ^^^** | **Wald chi-square (df=4)** | ***P* ^^^** | **Wald chi-square (df=4)** | ***P* ^^^** |
| **Antropometric** |  |  |  |  |  |  |  |  |  |  |
| Weight (kg) | 7.62 | .05 | 2.6 | .46 | 3.3 | .35 |  |  |  |  |
| BMI (kg/m2) | 7.01 | .72 | 2.3 | .51 | 2.3 | .51 |  |  |  |  |
| Waist circumference (cm) | 12.09 | .007 | 8.78 | .03 | 1.47 | .69 |  |  |  |  |
| **Body composition** |  |  |  |  |  |  |  |  |  |  |
| Visceral fat (cm^2^) | 12.19 | .007 | 4.06 | .26 | 3.5 | .32 |  |  |  |  |
| Fat percentage (%) | 12.90 | .005 | 6.96 | .07 | 5.96 | .11 |  |  |  |  |
| Muscle percentage (%) | 11.53 | .009 | 5.56 | .135 | 4.9 | .18 |  |  |  |  |
| **Laboratory** |  |  |  |  |  |  |  |  |  |  |
| HbA1c (mmol/mol) | 2.69 | .44 | 2.7 | .44 | 3.61 | .31 | 4.52 | .21 | 37.74 | <.001 |
| Gluc (mmol/l) | 2.84 | .42 | 1.82 | .61 | 8.23 | .04 | 65.19 | <.001 | 277.4 | <.001 |
| Chol (mmol/l) | 2.49 | .48 | 0.96 | .81 | 11.5 | .009 | 31.62 | <.001 | 79 | <.001 |
| HDL (mmol/l) | 0.76 | .86 | 4.17 | .24 | 3.72 | .29 | 14.65 | .005 | 44.08 | <.001 |
| LDL (mmol/l) | 1.77 | .62 | 0.52 | .91 | 9.94 | .02 | 35.5 | <.001 | 118.64 | <.001 |
| Trigly (mmol/l) | 0.37 | .95 | 0.16 | .98 | 0.86 | .84 | 8.26 | .082 | 303.35 | <.001 |
| ALAT (U/l) | 7.62 | .05 | 2.50 | .48 | 5.1 | .16 |  |  |  |  |
| Hs-CRP (mg/l) | 17.45 | <.001 | 1.08 | .78 | 2.6 | .46 |  |  |  |  |
| **Blood pressure** |  |  |  |  |  |  |  |  |  |  |
| Systolic (mmHg) | 5.59 | .13 | 4.75 | .19 | 6.44 | .09 | 7.59 | .11 | 9.43 | .05 |
| Diastolic (mmHg) | 5.04 | .17 | 2.91 | .41 | 3.29 | .35 | 4.47 | .35 | 9.38 | .05 |
| Pulse (BPM) | 0.29 | .96 | 0.51 | .92 | 0.63 | .89 | 5.48 | .24 | 2.3 | <.001 |

^^^ *P* value indicates the significance of the interaction between the variables over the four measurement points when the other variables used in the model are standardized
